# Childhood Adversity Moderates the Effects of *HTR2A* Epigenetic Regulatory Polymorphisms on Rumination

**DOI:** 10.3389/fpsyt.2019.00394

**Published:** 2019-06-14

**Authors:** Nora Eszlari, Peter Petschner, Xenia Gonda, Daniel Baksa, Rebecca Elliott, Ian Muir Anderson, John Francis William Deakin, Gyorgy Bagdy, Gabriella Juhasz

**Affiliations:** ^1^Department of Pharmacodynamics, Faculty of Pharmacy, Semmelweis University, Budapest, Hungary; ^2^NAP-2-SE New Antidepressant Target Research Group, Hungarian Brain Research Program, Semmelweis University, Budapest, Hungary; ^3^MTA-SE Neuropsychopharmacology and Neurochemistry Research Group, Hungarian Academy of Sciences, Semmelweis University, Budapest, Hungary; ^4^Department of Psychiatry and Psychotherapy, Kutvolgyi Clinical Centre, Semmelweis University, Budapest, Hungary; ^5^SE-NAP 2 Genetic Brain Imaging Migraine Research Group, Hungarian Brain Research Program, Semmelweis University, Budapest, Hungary; ^6^Division of Neuroscience and Experimental Psychology, Faculty of Biology, Medicine and Health, University of Manchester, Manchester, United Kingdom; ^7^Manchester Academic Health Sciences Centre, Manchester, United Kingdom; ^8^Greater Manchester Mental Health NHS Foundation Trust, Manchester, United Kingdom

**Keywords:** childhood stress, rumination, brooding, serotonin system, perseverative thought

## Abstract

The serotonin system has been suggested to moderate the association between childhood maltreatment and rumination, with the latter in its turn reported to be a mediator in the depressogenic effect of childhood maltreatment. Therefore, we investigated whether the associations of two epigenetic regulatory polymorphisms in the *HTR2A* serotonin receptor gene with Ruminative Responses Scale rumination and its two subtypes, brooding and reflection, are moderated by childhood adversity (derived from the Childhood Trauma Questionnaire) among 1,501 European white adults. We tested *post hoc* whether the significant associations are due to depression. We also tested the replicability of the significant results within the two subsamples of Budapest and Manchester. We revealed two significant models: both the association of methylation site rs6311 with rumination and that of miRNA binding site rs3125 (supposed to bind miR-1270, miR-1304, miR-202, miR-539 and miR-620) with brooding were a function of childhood adversity, and both interaction findings were significantly present both in the never-depressed and in the ever-depressed group. Moreover, the association of rs3125 with brooding could be replicated across the separate subsamples, and remained significant even when controlling for lifetime depression and the Brief Symptom Inventory depression score. These findings indicate the crucial importance of involving stress factors when considering endophenotypes and suggest that brooding is a more promising endophenotype than a broader measure of rumination. Transdiagnostic relevance of the brooding endophenotype and the potential of targeting epigenetic regulatory polymorphisms of *HTR2A* in primary and secondary prevention of depression and possibly of other disorders are also discussed.

## Introduction

Ruminative response style (rumination) is a passive and repetitive way of responding to distress and depressed mood, and it predicts future depression (1). It has two subtypes: the more maladaptive brooding and the less maladaptive reflection (2). Brooding means passive comparisons with unachieved standards, whereas reflection denotes purposeful problem-solving strategies (2).

It has been suggested that the depressogenic effect of childhood maltreatment is partly mediated by rumination (3). However, both retrospective studies (4, 5) and also a longitudinal one (6) indicates that this mediatory effect is carried by the brooding but not the reflection subtype of rumination, although one study found no evidence on the mediating effect of brooding between childhood maltreatment and adolescent internalizing or externalizing psychopathology (7). The relationship between childhood maltreatment and reflection is contradictory in itself, since two studies suggest their positive association (4, 6), but another one suggests no association between them (5). In contrast, brooding level is consistently predicted by childhood maltreatment (4, 6) and its all forms except for physical neglect (5).

The moderating role of the *5-HTTLPR* length polymorphism of the serotonin transporter gene in the effect of childhood maltreatment on rumination (8) indicates that the serotonin system seems to play a relevant role in rumination. In addition, tryptophan depletion in humans provokes cognitive inflexibility and a deficit in inhibitory control (9), both of which have been associated with rumination (10, 11), further supporting the involvement of serotonin function in rumination.

Among elements of the serotonin system, the neocortical serotonin 2A (5-HT_2A_) receptor appears to be a promising additional candidate with regard to rumination, since it mediates the effect of tryptophan depletion on response inhibition (12), and it has also shown alterations in radioligand binding and functional regulation by messenger RNA levels and by protein kinase A activity in the prefrontal cortex in several psychiatric disorders (13). Moreover, frontal 5-HT_2A_ binding has been associated with dysfunctional attitudes among patients in a major depressive episode (14, 15), and 5-HT_2A_ binding in the left dorsal prefrontal cortex has shown a positive correlation with the anticipatory worry subscale of harm avoidance (16). Dysfunctional attitudes and worry are both related to rumination (1, 10), consistent with a link between 5-HT_2A_ and rumination.

Early life stress can affect gene expression through epigenetic mechanisms including DNA methylation and microRNA (miRNA) expression (17). Therefore, in investigating the link between rumination-related phenotypes, childhood maltreatment and functioning of 5-HT_2A_, single nucleotide polymorphisms (SNPs) facilitating epigenetic regulation of the *HTR2A* gene encoding the receptor appear to be promising targets. Such regulatory SNPs have extensively been investigated in the background of rumination-related phenotypes. Increased *HTR2A* gene expression has been inconsistently linked to either the T allele of the methylation site T102C (rs6313) (18) SNP of exon-1 in the cortex (15), or to its C allele in the frontal lobe (19). Nevertheless, TC heterozygotes perform worse on the Wisconsin Card Sorting Task (WCST) than both homozygote groups (20, 21), and WCST performance correlates negatively with rumination (1). Rs6313 is in high linkage disequilibrium (LD) with another methylable SNP in the promoter region of the gene, rs6311 (-1438 A/G) (19, 22–24). Rs6313 T allele corresponds to rs6311 T allele (this latter one is measured on the positive strand of the DNA double helix, https://genome.ucsc.edu/) (25). Rs6311 also entails controversial phenotypic associations. Its T allele is related to an increased promoter function (15, 26–28) and mood disorders in one study (26) but unrelated to either major depression or bipolar depression according to meta-analyses (29, 30); nevertheless, its CC genotype (defined also on the positive strand) is the one that confers a risk for higher neuroticism, depression, and emotion-based coping strategies (31), all of which have been associated with rumination (1).

**Figure 1:**
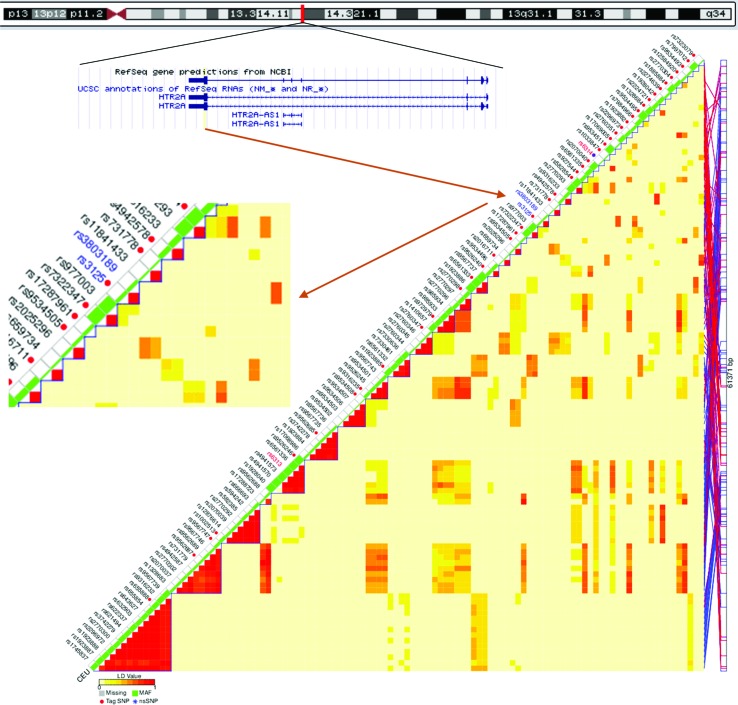
Position of rs3125 within the *HTR2A* gene on chromosome 13 (https://genome.ucsc.edu/, build hg19), and among tag single nucleotide polymorphisms (SNPs) of the gene in the HapMap CEU population (https://snpinfo.niehs.nih.gov/snpinfo/snptag.html). CEU = a population with Northern and Western European ancestry (https://www.sanger.ac.uk/resources/downloads/human/hapmap3.html); LD = linkage disequilibrium between each pair of SNPs, measured with r^2^; MAF = minor allele frequency.

In our present study, we investigated the associations of rumination with two regulatory SNPs from the two ends of the *HTR2A* gene. In addition to the promoter methylation site rs6311 (25), we chose the miRNA binding site rs3125 (https://snpinfo.niehs.nih.gov/snpinfo/snpfunc.html) residing in the 3′ UTR (untranslated region), the other regulatory side of the gene, according to hg19 database of the UCSC Genome Browser (https://genome.ucsc.edu/). Rs3125 is supposed to bind miR-1270, miR-1304, miR-202, miR-539, and miR-620 (https://snpinfo.niehs.nih.gov/cgi-bin/snpinfo/mirna.cgi?2_rs3125). It can be a tag SNP, representing its haploblock that consists of two SNPs (**Figure 1**), and it has been associated with depression among cardiac patients (32). We tested the main effect of these two SNPs, and their interactions with childhood adversity, on rumination and its two subtypes: brooding and reflection. In case of finding a significant effect, we tested whether or not it is mediated or moderated by depression. We hypothesize to reveal both main effects and interaction effects of the SNPs, based on the abundant main effect findings of *HTR2A* and the interaction effect literature of *5-HTTLPR*. As supplementary analyses, we ran the same models with *5-HTTLPR*.

## Methods

Our study, carried out in accordance with the Declaration of Helsinki, and being part of the NewMood (New Molecules in Mood Disorders) study funded by the European Union (Sixth Framework Program of the EU, LSHM-CT-2004-503474), was approved by the Scientific and Research Ethics Committee of the Medical Research Council, Budapest, Hungary, and by the North Manchester Local Research Ethics Committee, Manchester, United Kingdom.

### Participants

Subjects provided written informed consent, and they were recruited through advertisements and general practices from Budapest, Hungary, and through advertisements, general practices, and a website from Greater Manchester, UK. They received nothing for participation.

There were 1,501 adults (18–60 years old) from Budapest (*N* = 470) and Manchester (*N* = 1,031) that provided self-report questionnaire data about sex, age, and rumination, which reported to be of European white ethnic origin and were successfully genotyped for rs3125. None of them reported having had any relative participating in the study. Among these included participants, rs6311 was successfully genotyped in 469 subjects from Budapest and 1,017 from Manchester, and childhood adversity measure was provided by 468 subjects from Budapest and 1,030 from Manchester. Data on both rs6311 and childhood adversity were available in 467 subjects from Budapest, and 1,016 from Manchester. **Supplementary Figure 1** displays a flowchart on inclusion criteria and data availability.

### Phenotypic Measures

We measured rumination and its two subscales, brooding and reflection, with the 10-item Ruminative Responses Scale (RRS) (2). Five items of RRS belong to the brooding subscale, and five items to the reflection subscale, and scores on the two subscales add up to the rumination score. Brooding and reflection scores have a Pearson correlation of *r* = 0.487 (*N* = 1,501; *p* < 0.00001) in the combined sample, *r* = 0.308 (*N* = 470; *p* < 0.00001) in the Budapest subsample, and *r* = 0.521 (*N* = 1,031; *p* < 0.00001) in the Manchester subsample.

We used depression items plus the additional items from the Brief Symptom Inventory (BSI) (33) to measure current depressive symptoms. Each of the rumination scores and the BSI depression score was calculated as a weighted score: the sum of item scores divided by the number of completed items. Lifetime depression was measured by a question in the background questionnaire, and had been validated previously with face-to-face diagnostic interviews within a subpopulation, yielding a 91.7% sensitivity and 89.8% specificity (34).

Our childhood adversity measure (provided as **Supplementary Data**) was based on the Childhood Trauma Questionnaire (CTQ) (35), but is different from that in the specific items. Childhood adversity score means the sum score of four items regarding emotional and physical abuse and neglect, and two items about parental loss. We had validated this short childhood adversity measure previously with the 28-item CTQ within a subpopulation, yielding a significant Pearson* r* = 0.75 (*p* < 0.001) (34).

### Genotyping

By a genetic saliva sampling kit, subjects provided buccal mucosa cells personally or by post, from which genomic DNA was extracted according to Ref. (36). Rs3125 and rs6311 were genotyped with the Sequenom MassARRAY technology (www.sequenom.com, Sequenom, San Diego, CA, USA). Genotyping of *5-HTTLPR* has been detailed in one of our previous papers (37). All laboratory works were performed under the ISO 9001:2000 quality management requirements, and it was blinded with regard to phenotype.

### Statistical Analyses

We used Plink v1.07 (http://zzz.bwh.harvard.edu/plink/) to calculate Hardy–Weinberg equilibrium and allele frequencies, and, with the aid of individually written scripts in R (38), to run regression models. SPSS25 was used to perform χ^2^ or *t* tests, as appropriate, for descriptive statistics, and univariate general linear models for visualization purposes in the figures. In these general linear models, childhood adversity score was grouped into three categories: low (0–3); medium (4–6); and high (7 or more). Possible childhood adversity score ranges from 0 to 18, and in our study from 0 to 16. The same grouping to low, medium, and high scores had been applied in our previous study (39). In the general linear models run to visualize interaction effects, covariates were main effects of the respective SNP and childhood adversity, and the outcome variable had been controlled for the covariates detailed below, in a previous general linear model.

In our Plink linear regression models population, sex and age were covariates in all the analyses, and in case of each rumination subscale as the outcome, the other subscale was also a covariate. When testing an SNP × childhood adversity interaction effect, main effects of both the SNP and childhood adversity were included as covariates in the model.

As primary analyses, 36 regression equations were run in the Budapest + Manchester combined sample: with either of the two SNPs as predictors, on rumination, brooding and reflection as the outcome variable, testing either a main effect or an interaction effect of the SNP, in additive, dominant, and recessive models.

QVALUE v1.0 (40) was used to calculate *q*-values of false discovery rate (FDR) for the *p*-values of these 36 tests, without robust method, with a 0 to 0.99 range of λ (by 0.05), and a bootstrap method to estimate the overall proportion of true null hypotheses, π_0_. We consider results with a *q*-value of ≤0.05 as significant after correction for multiple testing.

For these primary tests, power calculation was carried out with Quanto v1.2 (http://biostats.usc.edu/Quanto.html), at a type I error rate of 0.05, assuming an R_G_^2^ = 1% for the SNP in the main effect models, and, in case of interaction models, assuming an R_GE_^2^ = 0.5% for the gene–by–environment (GxE) interaction, R_G_^2^ = 0% for the genetic main effect, and based on coefficients of determination with childhood adversity, an R_E_^2^ = 0.066 for rumination, 0.0756 for brooding and 0.0276 or 0.0282 (as appropriate for the respective sample size) for reflection.

For significant findings among the 36 primary tests, we tested possible mediating effects in secondary (*post hoc*) analyses as follows. First, we built the same model as that of the significant finding, with logistic regression on lifetime depression and linear regression on BSI depression as the outcome. Second, we ran the same model again, on the rumination variable as the outcome and including both depression variables as additional covariates. Third, we ran the model on both depression variables separately, including the rumination variable as an additional covariate. To control for the overfitting of these models due to the several covariates, 100,000 permutations were run on the relevant term in each mediation model. To speed up analyses, permutation was run in Plink v1.90b6.9 (4^th^ March, 2019; https://www.cog-genomics.org/plink/1.9/). A label-swapping, max(T) permutation procedure was applied, yielding an empirical *p*-value for the regression term.

Besides mediating, moderating effects of depression were also tested *post hoc* for the significant findings, by running the same model separately in those who reported lifetime depression (ever-depressed) and in those who did not (never-depressed).

Significant findings were *post hoc* tested whether holding true in the separate Budapest and Manchester subsamples, by the same models described above but without population as a covariate.

To test effects of the *5-HTTLPR* length polymorphism residing within the promoter region of the serotonin transporter gene, its main effects and interaction effects with childhood adversity were tested within the combined sample in a manner similar to *HTR2A* testing, as supplementary analyses.

In all *post hoc* analyses, and also in results of descriptive statistics and supplementary analyses, the threshold for significance was *p* ≤ 0.05, and for trend, it was *p* ≤ 0.10.

## Results

### Descriptive Statistics

For frequencies or means of the variables, see **Table 1** and **Supplementary Table 1**. We can see that the two subsamples significantly differ from each other in all variables except for rs6311 and *5-HTTLPR* genotype frequencies.

Both *HTR2A* SNPs are in Hardy–Weinberg equilibrium in the combined sample and in the Manchester and Budapest subsamples (rs3125 has a *p* = 0.909 in the combined sample, *p* = 0.616 in Budapest, and *p* = 0.607 in Manchester; and rs6311 yields a *p* = 0.163 in the combined sample, *p* = 0.704 in Budapest, and *p* = 0.051 in Manchester). For rs3125, C is the minor allele, with an allele frequency of 0.1289. For rs6311, T is the minor allele, yielding an allele frequency of 0.4078.

**Table 1:** Descriptive statistics for the combined Budapest + Manchester sample and the two subsamples.

		Budapest + Manchester		Budapest		Manchester		Difference between Budapest and Manchester	
		Frequency	%	Frequency	%	Frequency	%	χ^2^	p
**Sex**	**Male**	371	24.7%	88	18.7%	283	27.4%	13.209	0.0003
	**Female**	1,130	75.3%	382	81.3%	748	72.6%		
**Lifetime depression**	**Not reported**	824	54.9%	370	78.7%	454	44.0%	156.889	<0.00001
	**Reported**	677	45.1%	100	21.3%	577	56.0%		
***HTR2A*** ** rs3125**	**CC**	24	1.6%	6	1.3%	18	1.7%	8.678	0.013
	**CG**	339	22.6%	85	18.1%	254	24.6%		
	**GG**	1,138	75.8%	379	80.6%	759	73.6%		
***HTR2A*** ** rs6311**	**TT**	234	15.7%	83	17.7%	151	14.8%	2.589	0.274
	**TC**	744	50.1%	223	47.5%	521	51.2%		
	**CC**	508	34.2%	163	34.8%	345	33.9%		
		**Mean**	**S.E.M.**	**Mean**	**S.E.M.**	**Mean**	**S.E.M.**	**t**	**p**
**Age**		32.823	0.2747	30.315	0.4925	33.967	0.3249	−6.244	<0.00001
**Rumination score**		2.174	0.0151	1.986	0.0209	2.259	0.0192	−9.601	<0.00001
**Brooding score**		2.197	0.0178	1.954	0.0250	2.308	0.0224	−10.574	<0.00001
**Reflection score**		2.150	0.0172	2.019	0.0267	2.210	0.0217	−5.543	<0.00001
**Childhood adversity score**		3.392	0.0895	2.801	0.1365	3.660	0.1136	−4.838	<0.00001
**BSI depression score**		0.900	0.0244	0.540	0.0283	1.063	0.0319	−12.268	<0.00001

*χ* ^2^, Pearson *χ* ^2^; S.E.M., standard error of mean; BSI, Brief Symptom Inventory.

**Table 2** demonstrates that there is a significant gene-environment correlation in case of rs3125 and childhood adversity score in the combined sample and in Manchester.

**Table 2:** Effect of each *HTR2A* single nucleotide polymorphism (SNP) as predictor, for childhood adversity as outcome, in linear regression models.

		Additive model		Dominant model		Recessive model	
		Beta	*P*-value	Beta	*P*-value	Beta	*P*-value
rs3125	Budapest + Manchester	0.422	0.024	0.544	0.008	−0.338	0.630
	Budapest	0.182	0.561	0.275	0.424	−0.677	0.575
	Manchester	0.494	0.032	0.629	0.014	−0.286	0.739
rs6311	Budapest + Manchester	0.021	0.869	0.048	0.797	−0.006	0.979
	Budapest	−0.122	0.529	−0.483	0.090	0.338	0.343
	Manchester	0.102	0.547	0.302	0.205	−0.174	0.584

Sex and age (and in the combined sample also population) were covariates. The minor, effect allele is C for rs3125, and T for rs6311. Significant findings are marked with bold.

Among the 36 primary analysis models, those that test only the main effect of the SNP, without interaction term, have a 97.16% and a 97.28% power to detect it in case of rs6311 and rs3125, respectively. In case of testing the interaction effect with childhood adversity, rs6311 has 80.55% power for rumination, 80.95% for brooding, and 78.98% for reflection. Rs3125, in interaction with childhood adversity, has 80.94% power for rumination, 81.34% for brooding, and 79.36% for reflection. These findings indicate sufficient power to detect the tested genetic effects.

### Effects of *HTR2A* rs3125 and rs6311 on Rumination and Its Subtypes

**Figure 2:**
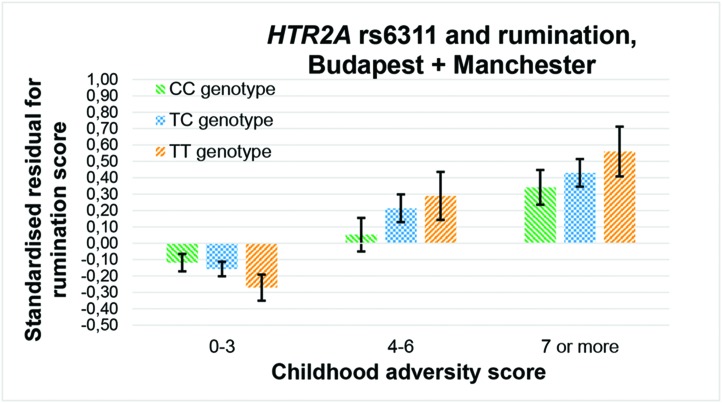
Interaction effect of childhood adversity and *HTR2A* rs6311 genotype on rumination score in a general linear model performed with visualization purposes.

**Figure 3:**
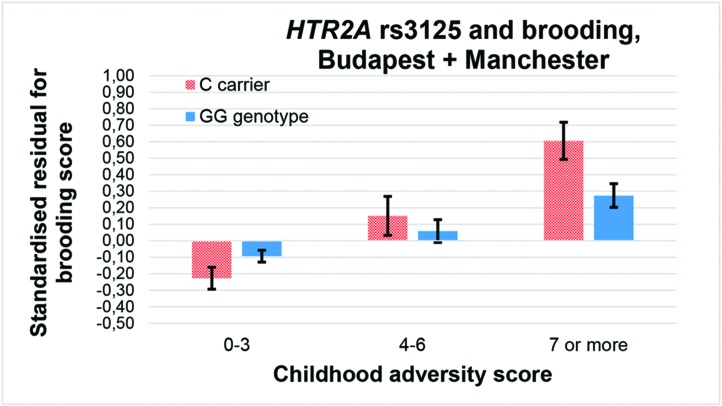
Interaction effect of childhood adversity and *HTR2A* rs3125 genotype on brooding score in a general linear model performed with visualization purposes.

Among the 36 equations (**Table 3**), only the rs6311 × childhood adversity interaction on rumination in an additive model (**Figure 2**), and the rs3125 × childhood adversity interaction on brooding in both an additive and a dominant (**Figure 3**) model, proves to be significant after correction for multiple testing.

**Table 3:** Effect of each *HTR2A* SNP as predictor, for each rumination variable as outcome, in linear regression models.

	Outcome	SNP	Additive model			Dominant model			Recessive model		
			Beta	*P*-value	Q-value	Beta	*P*-value	*Q*-value	Beta	*P*-value	*Q*-value
Main effect of SNP	Rumination	rs3125	0.041	0.180	0.114	0.061	0.068	0.085	−0.133	0.245	0.116
		rs6311	0.001	0.953	0.212	0.011	0.724	0.176	−0.014	0.727	0.176
	Brooding	rs3125	0.010	0.747	0.176	0.014	0.686	0.176	−0.020	0.871	0.199
		rs6311	0.020	0.367	0.124	0.030	0.348	0.124	0.019	0.640	0.176
	Reflection	rs3125	0.035	0.278	0.116	0.053	0.132	0.114	−0.127	0.291	0.116
		rs6311	−0.019	0.400	0.124	−0.018	0.570	0.166	−0.034	0.404	0.124
SNP × childhood adversity interaction	Rumination	rs3125	0.011	0.173	0.114	0.011	0.203	0.116	0.020	0.582	0.166
		rs6311	0.015	0.013	0.035	0.017	0.046	0.073	0.022	0.042	0.073
	Brooding	rs3125	0.028	0.001	0.006	0.030	0.001	0.006	0.036	0.345	0.124
		rs6311	0.008	0.227	0.116	0.010	0.282	0.116	0.010	0.394	0.124
	Reflection	rs3125	−0.015	0.091	0.091	−0.017	0.075	0.085	−0.013	0.729	0.176
		rs6311	0.009	0.147	0.114	0.010	0.286	0.116	0.016	0.186	0.114

Population, sex, and age were additional predictors in all models. In case of brooding or reflection as outcome, the other subscale was also a predictor. In the interaction models, main effects of both the respective SNP and childhood adversity were included as additional predictors. Findings having a q ≤ 0.05, thus surviving the correction for multiple testing, are marked with bold. The minor, effect allele is C for rs3125, and T for rs6311. SNP, single nucleotide polymorphism.

### Moderating Role of Depression in the Effect of the Investigated *HTR2A* Variants on Rumination Phenotypes

The rs6311 × childhood adversity interaction on rumination in an additive model remains significant both in ever-depressed (*N* = 670; β = 0.015; *p* = 0.045) and in never-depressed (*N* = 813; β = 0.019; *p* = 0.028) participants, indicating that depression history does not moderate this effect.

Similarly, the rs3125 × childhood adversity interaction on brooding remains significant in both ever-depressed (*N* = 676; additive β = 0.026; *p* = 0.022; dominant β = 0.029; *p* = 0.023) and never-depressed (*N* = 822; additive β = 0.032; *p* = 0.034; dominant β = 0.033; *p* = 0.033) participants.

### Mediating Role of Depression in the Found Effects

To test the possible mediating role of depression in the rs6311 × childhood adversity interaction effect on rumination and, vice versa, the possible mediating role of rumination in the same genetic effect on depression, the following prerequisites have to be met. Depression phenotypes have to show a significant positive correlation with rumination, and have to be associated with the rs6311 × childhood adversity interaction term in the same direction as rumination does. Rumination indeed shows a positive association with lifetime depression (*N* = 1,483; *t* = −18.304; *p* < 0.00001; with rumination means of 1.944 in the never-depressed and 2.451 in the ever-depressed group) and with BSI depression (*N* = 1,482; Pearson *r* = 0.570; *p* < 0.00001). However, the rs6311 x childhood adversity additive model does not yield a significant effect on either lifetime depression (*N* = 1,483; odds ratio (OR) = 1.022; *p* = 0.419), or BSI depression (*N* = 1,482; β = −0.001; *p* = 0.894), therefore we cannot test the possible mediating role of depression in this genetic effect on rumination. Nevertheless, including both depression phenotypes as covariates, the rs6311 × childhood adversity interaction on rumination in an additive model becomes more significant but with almost the same effect size (*N* = 1,482; β = 0.014; *p* = 0.005; empirical *p* = 0.005) as can be seen without controlling for depression (**Table 3**). This strengthening of significance may be due to the confounding effect of depression in the common variance of rumination and rs6311 × childhood adversity.

Prerequisites of testing the mediating role of depression in the significant rs3125 × childhood adversity effect on brooding are met. Brooding has a significant positive association with both lifetime depression (*N* = 1,498; *t* = −18.896; *p* < 0.00001; with means of 1.920 in the never-depressed and 2.534 in the ever-depressed group) and BSI depression (*N* = 1,497; Pearson *r* = 0.618; *p* < 0.00001). The rs3125 × childhood adversity interaction effect that has been found significant on brooding, is a trend on both lifetime depression (*N* = 1,498; additive OR = 1.081; *p* = 0.070; dominant OR = 1.087; *p* = 0.064) and BSI depression (*N* = 1,497; additive β = 0.024; *p* = 0.067; dominant β = 0.026; *p* = 0.060). All these associations enabled us to test mediating effects. Including both depression phenotypes as additional covariates, the rs3125 × childhood adversity interaction effect on brooding remains significant at a nominal *p* ≤ 0.05 level in both additive (β = 0.018; *p* = 0.015; empirical *p* = 0.015) and dominant (β = 0.019; *p* = 0.017; empirical *p* = 0.017) models. However, including brooding as an additional covariate, the effect of the rs3125 × childhood adversity interaction term considerably weakens on both lifetime depression (additive OR = 1.035; *p* = 0.449; empirical *p* = 0.453; dominant OR = 1.038; *p* = 0.430; empirical *p* = 0.431) and BSI depression (additive β = 0.005; *p* = 0.660; empirical *p* = 0.659; dominant β = 0.006; *p* = 0.600; empirical *p* = 0.600).

### Replicability of Significant Findings in the Separate Budapest and Manchester Subsamples

**Figure 4:**
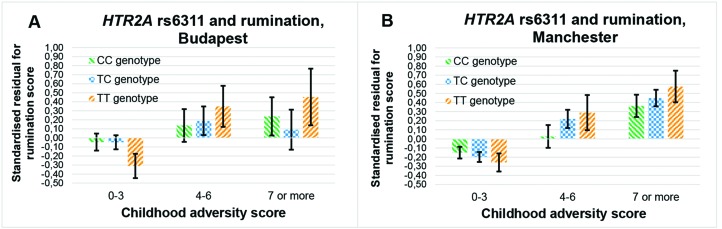
Interaction effect of childhood adversity and *HTR2A* rs6311 genotype on rumination score in a general linear model performed with visualization purposes in the Budapest **(A)** and Manchester **(B)** subsamples.

The rs6311 × childhood adversity additive model on rumination is not replicable, since it is not significant in Budapest (β = 0.009; *p* = 0.309), but it is significant in Manchester (β = 0.015; *p* = 0.050) (**Figure 4**).

**Figure 5:**
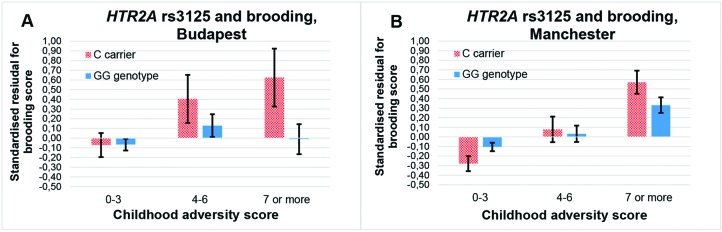
Interaction effect of childhood adversity and *HTR2A* rs3125 genotype on brooding score in a general linear model performed with visualization purposes in the Budapest **(A)** and Manchester **(B)** subsamples.

The rs3125 × childhood adversity effect on brooding can be replicated, because it is significant in both Budapest (additive β = 0.042; *p* = 0.026; dominant β = 0.042; *p* = 0.029) and Manchester (additive β = 0.024; *p* = 0.016; dominant β = 0.026; *p* = 0.016) (**Figure 5**).

### Effects of *5-HTTLPR* on Rumination and Its Subtypes

**Supplementary Table 2** demonstrates that *5-HTTLPR* is not associated with childhood adversity, entailing that there is no gene-environment correlation in its case.

**Supplementary Table 3** shows that *5-HTTLPR* is not associated with rumination, brooding or reflection, either in main effect or in interaction with childhood adversity.

## Discussion

We have demonstrated that both of two *HTR2A* polymorphisms related to two distinct epigenetic regulatory mechanisms exert a significant effect on current adult rumination as a function of childhood stress, independently of current and lifetime depression status. Both the methylation site rs6311 and the miRNA binding site rs3125 appear to contribute to the endophenotypic manifestation of rumination, and these findings also support the need to consider the role of childhood stress when considering endophenotypes. Moreover, the impact of the rs3125 × childhood adversity interaction on brooding could be replicated in Budapest and Manchester, remained significant when controlling for depression, and also fully mediated the weak effect of the same interaction term on depression. Thus, rs3125 and brooding show a stronger contribution to the endophenotypic manifestation of rumination phenotypes than rs6311 and a broader measure of rumination that encompasses the reflection subtype in addition to the brooding subtype. Rs3125 and childhood adversity may also convey a transdiagnostic relevance to the brooding endophenotype.

### *HTR2A* Effect on Adulthood Rumination Phenotypes Depends on the Level of Childhood Adversity, Independently of History of Depression

Contrary to former findings concerning *HTR2A* and rumination-related phenotypes (20, 21, 31), in our study, we found no main effect of any SNP in itself on rumination, but only an interaction with childhood adversity proved to be significant, similarly to the *5-HTTLPR* × childhood maltreatment interaction effect on rumination reported by Antypa and Van der Does (8). In line with this, our former results with *5-HTTLPR* and childhood adversity have also demonstrated the role of early tuning of the serotonin system in adulthood stress reactivity leading to depression (39). Although our present study did not find any significant association between *5-HTTLPR* and rumination itself, a possible mediating link between the detrimental impact of childhood maltreatment and serotonin transporter gene function may be regulation by epigenetic mechanisms. Epigenetic alterations, which are heritable and environmentally modifiable, denote reversible modifications to the genome, and they engender alterations in gene expression patterns in a cell-type specific manner (41). Early life stress has been proposed to have long-term consequences on brain structure and mental health outcomes *via* epigenetic mechanisms including methylation and miRNA expression, which were found to influence mental health phenotypes and serotonin transporter expression, respectively (17). An exposure during development may impact epigenetic states more broadly than later exposure (42), and especially miRNAs can totally switch expression during development but fine-tune it in adult tissues (43). In addition to former (but not present) findings with *5-HTTLPR*, our present results with *HTR2A* also underline the relevance of the serotonin system in the long-lasting effects of childhood stress that may be transmitted by epigenetic mechanisms.

In the promoter region of *HTR2A*, the cytosine at position -1439 can only be methylated in case of a G allele (or C allele if measured on the complementary, positive strand of DNA, as in our study) at the adjacent -1438 A/G (rs6311) polymorphism (19, 25, 44). Although methylation level of the promoter region of *HTR2A* was inversely related to the transient phenotype of antipsychotic use in the frontal lobe of schizophrenic and bipolar patients (19), its methylation in the placenta can entail long-term impacts on psychiatric phenotypes (44, 45). Previous contradictory results with rs6311 (26, 29–31) can be resolved by considering the moderating role of stress level, but recent stress has been suggested as much important as early stress in these terms. TT genotype denoted a risk for depression in case of a high childhood adversity level (46), and for a reduced heart rate variability only in case of a high level of recent stress (47). Heart rate variability has an inverse association with brooding (48), so our present results (see **Figure 2**) corroborate these former findings with another type of stress. As the T allele of rs6311 enhances expression (15, 26–28), and in accordance with that tryptophan depletion impairs response inhibition *via* an increased 5-HT_2A_ density within the right inferior frontal gyrus (12), we can hypothesize that a genetically heightened expression of *HTR2A* may make the level of rumination more dependent on 5-HT_2A_-mediated serotonin transmission, thus more sensitive to environmental impacts affecting the serotonin system, such as tryptophan depletion or stress.

Similarly, former results with the miRNA binding site rs3125 were inconclusive on bipolar disorder when stress level was not taken into account (49). On the other hand, the C allele appeared to increase risk of depression in cardiac patients (32) which can be considered a stressed group, corroborating our results also with the C allele as a risk variant for brooding only in case of high childhood stress (**Figures 3** and **5**). Rs3125 can bind to five different miRNAs (https://snpinfo.niehs.nih.gov/cgi-bin/snpinfo/mirna.cgi?2_rs3125) (50). Among them, miR-539 bound by the G allele of rs3125 showed a reduced expression in anterior cingulate cortex (ACC) in an animal model of chronic neuropathic pain (51). Although ACC has demonstrated a negative association in volume and resting state activity with rumination (52), and 5-HT_2A_ binding in ACC has been negatively related to treatment resistance in major depression (53), the exact role of rs3125 in *HTR2A* expression and the role of miRNA-regulated *HTR2A* expression in brooding have to be elucidated in the future. It should also be clarified whether childhood or recent timing of stress matters in the effect of rs3125 on brooding.

Both *HTR2A* × childhood adversity interaction effects were replicable separately in the ever-depressed and the never-depressed group, contributing to the endophenotypic manifestation of rumination. Namely, an endophenotype, by definition, should reside on the causal pathway between genetics and the disorder (54, 55), and rumination is not only augmented by a depressive episode even after recovery (56, 57), but it also predicts future onset of depression in never-depressed individuals (58). Our results point to the role of genetics in these associations and thus complete the picture with endophenotypic features, highlighting that *HTR2A* affects rumination not only in or after a depressive episode, but equally in those who have not yet developed or will never develop the disorder. Since high rumination constitutes a risk for depression, screening never-depressed people based on *HTR2A* rs3125 and rs6311 genotypes and levels of childhood adversity and rumination may once be part of a comprehensive picture in the primary prevention of depression. Similar screening in ever-depressed people could similarly aid relapse prevention as well as decision on pharmacotherapeutic and psychotherapeutic intervention.

### Effect of *HTR2A* rs3125 × Childhood Adversity on Brooding, a Possible Transdiagnostic Endophenotype Spanning European Populations

Although rumination score has shown endophenotypic features in our study, in that its association with rs6311 can be replicated both in the ever-depressed and the never-depressed group, it also seems multifaceted and can be further deconstructed to even simpler endophenotypes (59). Brooding may be its more useful subtype in primary and secondary depression prevention than the broader measure of rumination that includes also reflection. Indeed, reflection has not shown any association with any of the *HTR2A* polymorphisms in our present results.

Brooding may be a more useful endophenotype first because the rs3125 × childhood adversity interaction on brooding is replicable in Budapest and Manchester, while the rs6311 × childhood adversity interaction on rumination is significant only in Manchester but not in Budapest. Robustness of GxE (gene–by–environment) results on brooding is remarkable also because participants from Manchester have higher scores on childhood adversity and brooding, have a lower frequency of rs3125 GG genotype, and are more depressed than participants from Budapest (**Table 1**). Since our subsamples are not at all representative for the two populations in the variables of interest, further investigation is needed regarding the relative cross-population robustness of these genetic associations with brooding and the broader measure of rumination compared to each other.

Second, because the effect of *HTR2A* on brooding seems more relevant in depression than its effect on more broadly measured rumination. While the rs6311 × childhood adversity interaction term is not associated with depression at all, the rs3125 × childhood adversity interaction term has a trend effect on depression, which disappears if controlling for brooding. Brooding, however, remains associated with rs3125 × childhood adversity even if controlling for depression. All of these results point to a stronger association of rs3125 with brooding than with depression and suggest that rs3125 contributes to brooding as an endophenotype (59) that confers a risk for depression.

The investigated *HTR2A* genotypes exert an effect only on the broad measure of rumination and its brooding subtype but not on reflection. Brooding, being a more maladaptive subtype of rumination than reflection (2), may be a closer construct to those phenotypes previously having shown an association with 5-HT_2A_ or *HTR2A*: response inhibition deficit (12), perseverative errors on WCST (21), worry (16), dysfunctional attitudes (14, 15), neuroticism and emotion-based coping strategies (31). Moreover, our genetic findings with *HTR2A* × childhood adversity on rumination, brooding, and depression underline and expand previous results that only brooding but not reflection is important in the depressogenic effect of childhood maltreatment (4–6).

Further research is needed to clarify the origin of difference between miRNA binding and methylation of *HTR2A* in contributing to the endophenotypic manifestation of either brooding or the broader measure of rumination. Although considerable evidence underscores the interconnectedness of miRNA function and DNA methylation in gene regulation (60), also pointing to the precedence of miRNA functioning over DNA methylation in neuronal differentiation (61) and possibly in haloperidol effects (62), the exact relationship of these two types of epigenetic regulation regarding particularly *HTR2A* expression needs to be clarified.

Nevertheless, we can suggest that rs3125 can be a more useful biomarker in primary and secondary depression prevention than rs6311.

The fact that the rs3125 × childhood adversity interaction effect on brooding explains the same interaction effect on depression but is not explained by depression, implies that it goes beyond depression and may play a role in the potential transdiagnostic relevance of the brooding endophenotype. It may be part of the endophenotype conveying a risk for the disorders having been linked to both rumination and *HTR2A*, such as alcohol abuse (63–65), binge eating (66, 67) and obsessive-compulsive disorder (68–70). Transdiagnostic relevance of specifically the brooding subtype of rumination has already been suggested in obsessive-compulsive disorder and generalized anxiety disorder among unipolar depressed patients (68). Usefulness of *HTR2A* rs3125, childhood adversity and brooding in the primary and secondary prevention of all these disorders should be revealed in future studies.

### Limitations

Our study has several limitations which must be stated. Our statements about the effect of childhood adversity on adulthood rumination and the moderating role of genetic variants in this effect could be proven only in case of a longitudinal study design. However, our design being cross-sectional, we can draw conclusions only about associations between childhood stress and adulthood phenotype. A longitudinal design could similarly aid in clarifying the causal roles of rumination and depression in each other.

Furthermore, childhood stress was assessed retrospectively, and only by self-report of our subjects, but not ascertained by other informants. Thus assessment of childhood adversity is subject to memory, recall bias and also state-dependent recall, and could be biased by voluntary distortions related to psychiatric and personality disorders. Similarly, lifetime depression assessment was based on self-report but not corroborated by actual previous disease history.

Since we found a gene-environment correlation between rs3125 and childhood adversity both in the combined sample and in Manchester, it was crucial to replicate our rs3125 interaction findings in Budapest, and to include main effects of gene and environment as covariates in these interaction models. Our rs3125 × childhood adversity finding on brooding could be replicated also in Budapest, underlining that it was not, or not only due to the gene-environment correlations.

In the present study, we did not consider the behavior of individual items with respect to the applied scale scores, but treated them as equivalently weighted in adding up to the respective score. In the future, item-response analyses should argue for or against this equivalent ponderation of the individual items.

Another limitation of our study is the low number of SNPs within the investigated gene. Tagging *HTR2A* with more SNPs, or broadening our scope to rare variants, structural variants, copy number variants or other length polymorphisms, besides variants with a more diverse spectrum of annotation types (such as amino acid change as an additional type of consequence on gene functioning), will be able to provide a deeper insight into the role of *HTR2A* gene in the association of childhood stress and rumination. The same concept can then be applied in hypothesis-free genome-wide investigations, going beyond candidate genes.

Rumination was measured only by RRS, a self-report questionnaire that asks people to rate themselves related to when they feel depressed. Other rumination measurements would also be worth investigations with regard to *HTR2A*.

Stress was defined only as childhood stress. A more detailed picture would be gained by targeting the possible role of other types of stress, such as recent stress or medical conditions.

Our study population is limited to European white participants. To strengthen clinical relevance of our present results, future studies should confirm the same associations in other ethnicities.

Future studies should measure actual epigenetic markers that are supposed to mediate between the revealed GxE interactions and rumination phenotypes.

### Conclusions and Future Directions

Both of the investigated genetic variations involved in transmitting two distinct epigenetic regulatory mechanisms, promoter DNA methylation and miRNA binding in the 3′ UTR, acting on *HTR2A* gene, contribute to the endophenotypic manifestation of rumination, in that their effects can be detectable not only in depression but before or without the emergence of depression. Potentials of targeting *HTR2A* genetics in depression prevention deserve further studies. Our results on action of *HTR2A* in rumination phenotypes also underscore the need to include childhood adversity assessment in these possible prevention strategies, and, more broadly, to include stress or other environmental factors in considering endophenotypes, especially in case of polymorphisms conveying epigenetic impacts. Brooding seems a more promising candidate endophenotype in mediating vulnerability to depression than rumination measured more broadly, since its association with *HTR2A* can be replicated across two different European populations, and mediates the same genetic association with depression. The effect of rs3125 on brooding, depending on childhood adversity level, may have a wider transdiagnostic relevance for other disorders in which brooding may be important such as obsessive-compulsive disorder.

## Data Availability Statement

The datasets generated for this study are available on request to the corresponding author.

## Ethics Statement

Our study, carried out in accordance with the Declaration of Helsinki, was approved by the Scientific and Research Ethics Committee of the Medical Research Council, Budapest, Hungary, and by the North Manchester Local Research Ethics Committee, Manchester, United Kingdom. Subjects provided written informed consent.

## Author Contributions

JD, GB, IA, GJ, RE, and XG designed the study. GJ and XG performed data collection. NE, PP, and DB undertook statistical analyses. NE managed the literature search and wrote the first draft of the manuscript. All authors contributed to manuscript revision, read, and approved the submitted version.

## Funding

This study, as part of NewMood, was supported by the Sixth Framework Program of the European Union (LSHM-CT-2004-503474). Moreover, it was supported by the Hungarian Academy of Sciences (MTA-SE Neuropsychopharmacology and Neurochemistry Research Group), by the Hungarian Brain Research Program (grants KTIA_13_NAPA-II/14 and 2017-1.2.1-NKP-2017-00002, and with the Hungarian National Development Agency, the Hungarian Academy of Sciences and Semmelweis University, grant KTIA_NAP_13-2-2015-0001, MTA-SE-NAP B Genetic Brain Imaging Migraine Research Group), by the National Development Agency (KTIA_NAP_13-1-2013-0001), by the New National Excellence Program of The Ministry of Human Capacities (grants ÚNKP-16-3; ÚNKP-17-3-III-SE-2; ÚNKP-17-4-I-SE-8 and ÚNKP-18-4-SE-33), by TAMOP-4.2.1.B-09/1/KMR-2010-0001, and by the National Institute for Health Research Manchester Biomedical Research Centre. XG is recipient of the Janos Bolyai Research Fellowship of the Hungarian Academy of Sciences. None of the sponsors had any role in study design, data collection, analysis or interpretation, writing the report, or in the decision to submit the paper for publication.

## Author’s Note

Preliminary results of this work were presented by Nora Eszlari on the ECNP Workshop for Junior Scientists in Europe, 17–20 March 2016, Nice, France. It was published as an abstract in *European Neuropsychopharmacology* 26, S77–S78, with the title “Brooding subtype of rumination is modulated by the interplay between serotonin receptor 2A gene and childhood adversity” (72).

Results of this work appeared first and only in Nora Eszlari’s PhD dissertation (71). It is in line with the policy of Semmelweis University, and the dissertation can be accessed online (http://semmelweis.hu/wp-content/phd/phd_live/vedes/export/eszlarinora.d.pdf).

## Conflict of Interest Statement

RE has received consultancy fees from P1vital and Cambridge Cognition. IA has received grant support from AstraZeneca and Servier, honorarium for speaking from Lundbeck, and consultancy fees from Alkermes, Janssen, Lundbeck/Otsuka, and Servier. JD has performed speaking engagements, consultancy, and research for P1vital, Autifony, Bristol-Myers Squibb, AstraZeneca, Eli Lilly, Janssen-Cilag, Servier, and Schering Plough, with all fees paid to the University of Manchester. He also has share options in P1vital. The firms declared above have not influenced study design, data collection, analysis, interpretation, manuscript preparation, or decision to submit the paper for publication, in any manner.

The remaining authors declare that the research was conducted in the absence of any commercial or financial relationships that could be construed as a potential conflict of interest.
