# Supplementary material for: Childhood Adversity Moderates the Effects of HTR2A Epigenetic Regulatory Polymorphisms on Rumination
Source: Front Psychiatry. 2019 Jun 14;10:394. doi: 10.3389/fpsyt.2019.00394 (PMC6588047; doi:10.3389/fpsyt.2019.00394)
Supplement: Supplementary file 1 [file DataSheet_1.docx]

Childhood Adversity Moderates the Effects of HTR2A Epigenetic Regulatory Polymorphisms on Rumination

# Supplementary Data: Childhood adversity questionnaire used in our study

Here are some comments people have made about their childhood. We would like you to say **how much each statement is true for you**.

| **When I was growing up** | **Never True** | **Rarely True** | **Some-times True** | **Often true** | **Very Often True** |
| --- | --- | --- | --- | --- | --- |
| 1. I was happy |  |  |  |  |  |
| 2. I believe that I was abused or neglected |  |  |  |  |  |
| 3. People in my family looked out for each other |  |  |  |  |  |
| 4. My parents/guardians weren’t able to take care of me |  |  |  |  |  |

| **When I was growing up** | **YES** | **NO** |
| --- | --- | --- |
| 1. I lost my mother |  |  |
| 2. I lost my father |  |  |

# Supplementary Figures and Tables

## Supplementary Figures


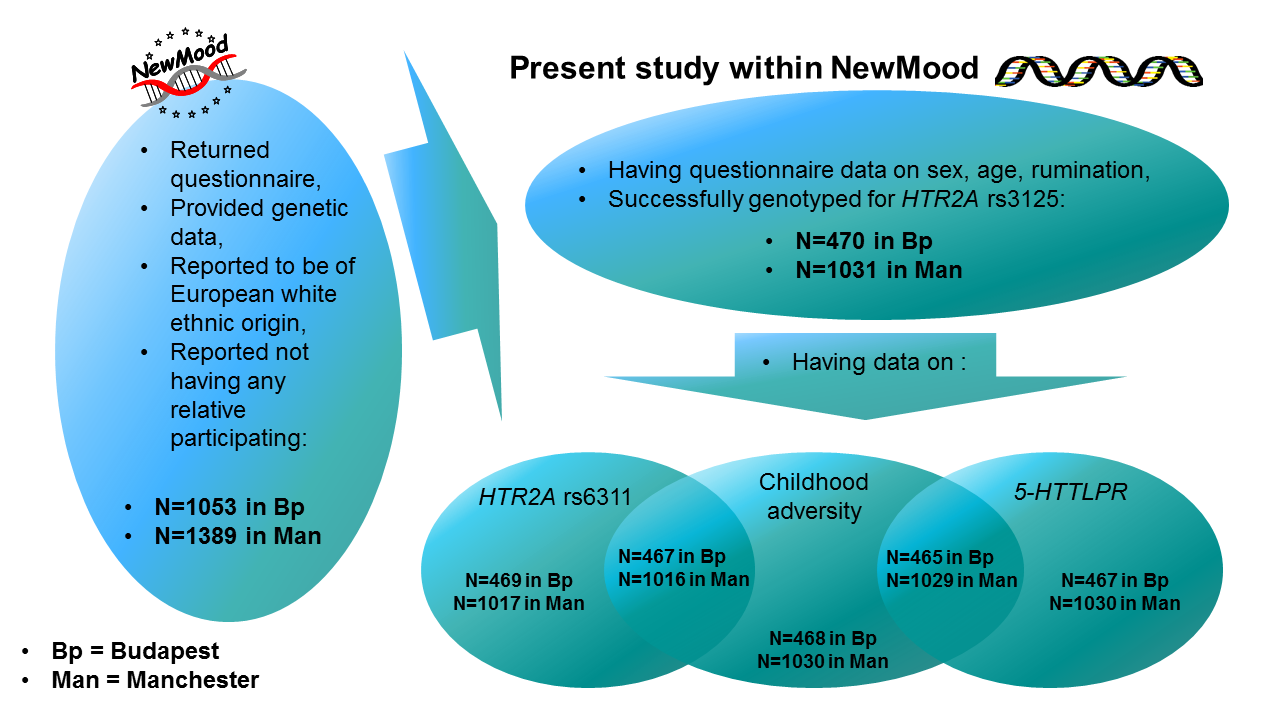


***Supplementary Figure 1*.** **Flowchart of the present study within the larger NewMood study.** Sample sizes are given according to the inclusion criteria, and for specific analyses within our study. Intersects of data availability according to the different variables of interest are also displayed.

## Supplementary Tables

***Supplementary Table 1.*** **Descriptive statistics of the serotonin transporter gene polymorphism *5-HTTLPR* for the combined Budapest + Manchester sample and the two subsamples.** *5-HTTLPR* = serotonin-transporter-linked polymorphic region; S = short allele; L = long allele; χ^2^ = Pearson χ^2^.

|  | **Budapest + Manchester** | | **Budapest** | | **Manchester** | | **Difference between Budapest and Manchester** | |
| --- | --- | --- | --- | --- | --- | --- | --- | --- |
|  | **Frequency** | **%** | **Frequency** | **%** | **Frequency** | **%** | **χ^2^** | **p** |
| **SS** | 272 | 18.2% | 72 | 15.4% | 200 | 19.4% | 3.672 | 0.159 |
| **LS** | 736 | 49.2% | 241 | 51.6% | 495 | 48.1% |  |  |
| **LL** | 489 | 32.7% | 154 | 33.0% | 335 | 32.5% |  |  |

***Supplementary Table 2.* Effect of *5-HTTLPR* as predictor, for childhood adversity as outcome, in linear regression models.** Sex and age (and in the combined sample also population) were covariates. The short allele is the minor, effect allele. No significant finding emerged. *5-HTTLPR* = serotonin-transporter-linked polymorphic region.

|  | **Additive model** | | **Dominant model** | | **Recessive model** | |
| --- | --- | --- | --- | --- | --- | --- |
|  | **Beta** | **P-value** | **Beta** | **P-value** | **Beta** | **P-value** |
| **Budapest + Manchester** | -0.045 | 0.722 | -0.211 | 0.262 | 0.166 | 0.470 |
| **Budapest** | 0.213 | 0.290 | 0.260 | 0.366 | 0.298 | 0.426 |
| **Manchester** | -0.150 | 0.346 | -0.421 | 0.080 | 0.110 | 0.699 |

***Supplementary Table 3.* Effect of *5-HTTLPR* as predictor, for each rumination variable as outcome, in linear regression models run in the Budapest + Manchester sample.** Population, sex and age were additional predictors in all models. In case of brooding or reflection as outcome, the other subscale was also a predictor. In the interaction models, main effects of both the respective SNP and childhood adversity were included as additional predictors. The short allele is the minor, effect allele. No significant finding emerged. *5-HTTLPR* = serotonin-transporter-linked polymorphic region.

|  |  | **Additive model** | | **Dominant model** | | **Recessive model** | |
| --- | --- | --- | --- | --- | --- | --- | --- |
|  | **Outcome** | **Beta** | **P-value** | **Beta** | **P-value** | **Beta** | **P-value** |
| **Main effect of *5-HTTLPR*** | **Rumination** | -0.019 | 0.353 | -0.045 | 0.139 | 0.004 | 0.905 |
|  | **Brooding** | -0.008 | 0.697 | -0.021 | 0.505 | 0.004 | 0.915 |
|  | **Reflection** | -0.013 | 0.559 | -0.028 | 0.376 | 0.001 | 0.985 |
| ***5-HTTLPR* x childhood adversity interaction** | **Rumination** | 0.004 | 0.473 | 0.014 | 0.102 | -0.007 | 0.497 |
|  | **Brooding** | 0.008 | 0.197 | 0.008 | 0.347 | 0.013 | 0.232 |
|  | **Reflection** | -0.003 | 0.610 | 0.008 | 0.413 | -0.021 | 0.056 |
